# Biomarkers in psychiatry: how close are we?

**DOI:** 10.3389/fpsyt.2012.00114

**Published:** 2013-01-07

**Authors:** Firas Kobeissy, Ali Alawieh, Stefania Mondello, Rose-Mary Boustany, Mark S. Gold

**Affiliations:** ^1^Division of Addiction Medicine, Department of Psychiatry, Center for Neuroproteomics and Biomarkers Research, University of FloridaGainesville, FL, USA; ^2^Department of Biochemistry, College of Medicine, American University of BeirutBeirut, Lebanon; ^3^Department of Neuroscience, University of MessinaMessina, Italy

**A commentary on**

**Systems biology, bioinformatics and biomarkers in neuropsychiatry**

by Alawieh, A., Zaraket, F., Li, J., Mondello, S., Nokkari, A., Razafsha, M., et al. (2012). Front. Neurosci. 6:187. doi: 10.3389/fnins.2012.00187

Until recently, there has been an ongoing worldwide quest in search for disease-specific molecular biomarkers in medicine. These biological molecules can allow for: a reliable and accurate disease diagnosis and prognosis, better understanding of pathogenesis and pathophysiological mechanisms, and for predicting disease progression and monitoring therapy. Furthermore, biomarkers can provide major opportunities for drug target identification which can ultimately translate into new therapeutic strategies with disease-modifying effects. Notably, all of these conditions are inherent characteristics of neuropsychiatric diseases.

Biomarker research has achieved great success in various clinical fields such as cardiovascular disease, hepatic disorders, neurotrauma leading to key markers including the discovery of troponin as marker for myocardial infarction, and 14-3-3 protein for Creuzfolt-Jacob Disease, S100β/UCH-L1/αII-spectrin for brain trauma (Hayes et al., 2011; Kobeissy et al., 2011; Mondello et al., 2011). However, in psychiatry this field is still lagging since no putative biomarker has yet made its way into clinical application (Schulenborg et al., 2006; Lescuyer et al., 2007).

Biological psychiatry research has been introduced as an attempt to draw psychiatry back to its biological roots in order to improve injury mechanisms and disease processes and its components. It has been well-understood today in clinical medicine that no promising accurate and definite disease diagnosis, therapy, and prognosis can be established without drawing back the clinical manifestation of the disease. Therefore, biological psychiatry is now focusing on the use of all available advanced molecular techniques that can allow for biomarker detection assisted by the afore-employed imaging and analysis techniques. Such approaches include the utilization of high throughput omics approaches such as: epigenetics, genomics, proteomics, lipidomics, and metabolomics studies (Robeva, 2010; Westerhoff, 2011). In addition, these methodologies rely on sophisticated computational-multi disciplinary field of systems biology utilizing advanced bioinformatics processing tools that can interpret the high throughput molecular omics data relevant to neuropsychiatric research. Among the ultimate aims of such discipline is the identification of novel sensitive and disease-specific biomarker(s).

The promise that systems biology can lead a progress in biological psychiatry returns to the very complex nature of psychiatric disorders. Such disorders involve multifactorial genetic and environmental interactions together with the dynamic nature of protein alterations affecting both cellular as well as structural changes on the neuronal levels. Therefore, assessing psychiatric disorders cannot be targeted at a single behavioral or cellular level but rather would require a holistic global approach that can assess different components of such disorders (Fang and Casadevall, 2011; Westerhoff, 2011). This can lead the inquiry into the roots of such disorders and identify new diagnostic and assessment biomarkers. However, the need for biomarker discovery and the implementation of systems biology techniques is not just because of the complexity of the disease. It is also an attempt to surmount the available diagnostic techniques such as DSM IV and ICD-10 that involve “subjective” checklist analysis of signs and symptoms of these diseases that causes frustration among most psychiatric practitioners (Linden, 2012; Tretter and Gebicke-Haerter, 2012).

Having been said, there has been a pronounced worldwide joint effort in the advancing of biomarker studies that is evident by the surge of research and review articles focusing on the application of systems biology, bioinformatics, and biomarkers in neuropsychiatry. These studies have included the use of high-throughput genomic, epigenetics, proteomic, metabolomics, and other—bioinformatic computational algorithms tools as well as the use of animal models, *in vitro and in vivo* tissue cultures and *in silico* models. These techniques have been applied on different aspects of neuropsychiatric disorders spanning: drug abuse, eating disorders, and other psychiatric disorders involving schizophrenia, bipolar disorder, and major depressive disorder etc. (Kobeissy et al., 2008; Avena, 2011).

The application of these techniques has provided several disease models of psychiatric diseases (Tretter and Gebicke-Haerter, 2012) that have moved research and therapy forward as with the dopamine agonist model of schizophrenia that we reviewed in a separate publication (Alawieh et al., 2012). However, success reported by using such techniques is still in its infancy due to the aforementioned complexity of psychiatric diseases as well as for other reasons. These include, on one hand, the limitations associated with these techniques coupled with “*mindset*” related to scientists and researchers that emphasizes on data discovery rather than data analysis and validation. This resulted in massive amount of data—majorly non-replicable and non-validated—with very low biological significance and clinical impact (Kraemer et al., 2002; Staner, 2006; Martins-De-Souza et al., 2011). Therefore, there is now an uprising need for the integrative and predictive analysis as well as validation of the available data collected to infer the biological significance relevant to psychiatry.

Finally, the field of biomarker discovery in psychiatry, taking advantage of systems biology approach and the available bioinformatics tools, is believed to yield several advantages including early diagnosis that is critical to psychiatric diseases and accurate criteria for disease, diagnosis, classification, and stratification. It can also allow for advanced personalized therapy and can act, if appropriately, validated as surrogate end points that can eliminate several limitations and greatly advance clinical research (Biomarkers Definitions Working Group, 2001; Zhang et al., 2010).
